# Hamstring muscle fibre typology is not associated with hamstring strain injury history or performance in amateur male soccer players: a retrospective magnetic resonance spectroscopy study

**DOI:** 10.5114/biolsport.2023.126663

**Published:** 2023-05-31

**Authors:** Joke Schuermans, Erik Witvrouw, Evi Wezenbeek, Eline Lievens

**Affiliations:** 1 Department of Rehabilitation Sciences, Faculty of Medicine and Health Sciences, Ghent University, Ghent, Belgium; 2 Department of Movement Sciences, Faculty of Medicine and Health Sciences, Ghent University, Ghent, Belgium

**Keywords:** Hamstring Strain Injury, Soccer, Muscle Fiber Type, Carnosine, Proton Magnetic Resonance Spectroscopy (^1^H-MRS)

## Abstract

Hamstring strain injuries (HSI) are still the most common injuries in soccer. Recent research has been focusing on the role of hamstring muscle morphology and architecture. The hamstring’s fibre type composition might play a role as well, but this has never been investigated in the light of HSI risk in an athletic population. The purpose of this study was to investigate the association between hamstring muscle fibre type, hamstring strain injury history (HSIH), performance and isokinetic strength in a population of amateur male soccer players. In this cross-sectional observational study, 44 male soccer players (22 with and 22 without HSIH) participated. The research consisted of a non-invasive fibre composition evaluation using proton magnetic resonance spectroscopy (^1^H-MRS), functional performance (evaluated by means of maximal jumping height, maximal sprinting speed and hamstring muscle strength endurance (single leg hamstring bridge testing)), and isokinetic strength testing. The results revealed that hamstring carnosine concentration demonstrated a high inter-individual variability within this soccer population and was not significantly associated with either HSIH or with any of the functional performance parameters. The only secondary outcome measure presenting a significant association with the intramuscular carnosine content was the hamstrings’ explosive strength production capacity, objectified by means of the time to peak torque (TPT), measured concentrically at an angular velocity of 240 degrees/second (°/s) during isokinetic strength testing. This TPT was significantly shorter in players presenting higher carnosine concentrations (p = 0.044). The findings indicate that in male amateur soccer players (1) the hamstrings have no distinct fibre type dominance and (2) fibre typology in this population does not relate to HSIH or performance.

## INTRODUCTION

Running-related hamstring strain injuries (HSI) continue to present high occurrences and reoccurrences in male soccer [1]. Next to the already repeatedly investigated athlete- and performance-related risk factors making the individual athlete more susceptible to HSI (age, injury history, (eccentric) muscle strength) [2–4], hamstring muscle fibre typology is believed to play a role in this [4]. Previous research on hamstring muscle fibre type composition suggested that the hamstrings are particularly susceptible to strain injuries, due to the fact that they consist of a high portion of fast twitch (FT) fibres, compared to other lower limb muscle groups [5–8]. Although fast twitch fibres have beneficial contractile characteristics (e.g. short time to peak tension) – facilitating the athlete’s explosiveness – they have a limited oxidative potential which limits both aerobic and repeated anaerobic exercise capacity due to lactate accumulation [9]. This could promote earlier fatigue onset and intramuscular acidification and increase their risk of strain injury during repeated high speed running [7, 10]. Moreover, FT fibres are more susceptible to structural damage induced by (eccentric) exercise compared to slow twitch (ST) fibres because of ultrastructural differences between both fibre groups, making FT fibres less resistant to strain (narrow Z-disks, lower molecular weight, lower isoform expression) [11]. Unlike the existing evidence and scientific background on fibre structure and architectural muscle-tendon features, studies on hamstring muscle fibre type composition are scarce and non-existent in the context of hamstring strain injury risk assessment, and have never been studied in athletic populations vulnerable to hamstring injury. One very recent study assessed the association between the soleus muscle’s carnosine content and the hamstring injury risk in professional male soccer players using a prospective design [12]. The results revealed that athletes with high intramuscular carnosine contents indicative for FT fibre dominance had a five-times greater risk of sustaining a hamstring injury compared to athletes presenting low carnosine contents, matching with slow twitch (ST) dominant fibre distributions. Although other researchers suggested the existence of an across-muscle phenotype with respect to fibre type proportions within one individual, indicative for a fairly homogeneous fibre type distribution across skeletal muscles within one subject [13], the role of the fibre type composition of the hamstring muscle unit itself in its particular vulnerability to strain injury has never been investigated.

The present study intends to examine to what extent hamstring muscle fibre type distribution is associated with hamstring injury history and athletic performance in a population of amateur male soccer players. This will be done using proton magnetic resonance spectroscopy (^1^H-MRS). This is a soft-tissue specific quantitative (metabolic and functional) MR technique that allows quantification of concentrations of particular metabolites. The dipeptide carnosine is a metabolite, of which the intramuscular concentrations have been established to be twice as high in fast twitch compared to slow twitch muscle fibres [14–15]. Previous research has validated this ^1^H-MRS technique as an alternative for invasive muscle sampling procedures with histochemical staining in the light of muscle fibre type identification [14].

The aim of the present study is to 1) identify the hamstring muscle’s fibre composition by means of ^1^H-MRS in a sample of amateur male soccer players; 2) assess the possible association between hamstring muscle fibre composition and hamstring strain injury history (HSIH); and 3) investigate the association between hamstring muscle fibre composition and athletic performance capacity, since both have been associated with HSI risk, which would imply both features to be mutually related as well.

## MATERIALS AND METHODS

Male soccer players, active in the amateur competition of Belgium, were addressed and informed as regards the purpose and content of this study via social media and by directly reaching out to some clubs affiliated to the head researcher’s private network. As this study intended to assess the association between hamstring fibre composition HSIH and athletic performance parameters, both soccer players with and without a (soccer-related) recent HSI history were recruited. Participants were selected as such, in order to end up with a sample with an equal representation of players with and without a history of HSI. This recent history of HSI needed to concern a non-contact soccer-related HSI occurring during training or match play, within the last two seasons. In order to be eligible for participation in the injury history group, these injuries needed to be confirmed by a medical doctor and needed to have prohibited the player from participating in regular training and match activities for at least one week. Participants categorized in this hamstring injury group needed to be fully recovered, participating in weekly training and competition sessions and free of any complaint or residual dysfunction/functional limitation at the time of testing. All male athletes between the age of 18 and 35 years (in order to exclude the presence of a potentially biasing effect of underlying age-related musculoskeletal disorders) were eligible for participation if they:

– Had been active in soccer competition for at least 5 years
– Had a weekly soccer exposure consisting of at least 2 training sessions and one match session
– Did not systematically participate in additional strength training or weight lifting in addition to their weekly soccer training (which might potentially influence intramuscular carnosine concentration)
– Had no recent injury (within the last 5 years) of orthopaedic surgery or severe musculoskeletal trauma necessitating them to stop soccer practice for a period longer than 3 months
– Were fully fit, free from any pain or complaint and ‘ready to compete’ at the time of participation
– Did not take any food supplements which might possibly influence the intramuscular carnosine content (beta-alanine) [15]
– Did not report being vegetarian or vegan, as these dietary measures might have an impact on the intramuscular carnosine concentration as well.

After being checked for eligibility to participate, willing candidates were contacted by phone and e-mail in order to verify their availability within the testing period (October, November and December 2020).

Ultimately, 44 male soccer players participated in this study, of whom 22 had and 22 lacked a history of HSI (matched controls). Participants within the control group were matched with participants in the HSIH group by means of age, height and weight. Details on anthropometric features and hamstring injury history can be seen in Table 1. Within the injury group, injuries were located at the level of the proximal myotendinous junction of the BF_LH_ in 40% of cases (mostly with involvement of the common tendon with the semitendinosus (SemiT)), at the level of the BF_LH_ muscle in 25% of cases and at the level of the SemiT muscle in 35% of cases. These details were gathered by means of a participant’s self-report, with additional availability of conforming medical imaging. Average time since the last HSI was 12.8 (± 9.3) months, and most of the candidates reported having sustained this HSI in the dominant leg (73%).

**TABLE 1:** Participant Details.

	HSI History group (n = 22)	Control group (n = 22)
Age (years, mean ± SD)	24.95 ± 3.47	22 ± 1.57
Bodyweight (kilograms, mean ± SD)	78.32 ± 8.80	74.98 ± 6.73
Height (meters, mean ± SD)	1.81 ± 0.06	1.81 ± 0.06
Time passed since last HSI (months, mean ± SD)	12.8 ± 9.3	/
Dominant leg involvement (%)	73	/

Note: HSI, Hamstring Strain Injury; n, sample size; SD, Standard Deviation; %, Percentage

Eligible candidates were invited to the Ghent University Hospital Campus to take part in a comprehensive testing procedure consisting of (1) proton magnetic resonance spectroscopy (^1^H-MRS) for quantification of the hamstring’s intramuscular carnosine content (measured in the SemiT) and (2) a functional performance and isokinetic strength testing battery.

This study was approved by the Ethics Committee of the Ghent University Hospital (approval number B670201941764, dd. 18/11/2019).

Each participant signed an informed consent form prior to participation in this study’s testing series. The duration of the entire testing procedure was approximately 1.5 hours per participant. After completing an extensive administrative survey regarding their athletic experience/background and (hamstring) injury history, participants were systematically submitted to the MRS testing procedure first, followed by the performance and isokinetic strength testing procedures. This standardized testing sequence was chosen as we wanted to collect baseline resting state MR images and spectroscopy spectra, in order to avoid bias induced by the potential influence of mechanical and metabolic muscle activation on the ^1^H-MRS characteristics.

A particular, soft-tissue-specific, MR sequence (3 Tesla (T) whole body MRI scanner (Prisma, Siemens)) was used in order to acquire hamstring muscle fibre architecture-related data in this study (point resolved spectroscopy sequence (PRESS)) [16]. Details on muscle fibre composition were gathered using a proton spectroscopy (proton magnetic resonance spectroscopy (^1^H-MRS)) sequence measuring the muscle tissue’s carnosine content, which provided the opportunity to make assumptions regarding the muscle’s fibre type dominance based on the intramuscular carnosine content, relative to the content of a water reference [12, 17–18]. Prior to this spectroscopy sequence, coronal and transverse plane T1 weighted images were taken for subsequent voxel positioning in the semitendinosus muscle. This spectroscopy sequence was systematically run on a homogeneous muscle tissue voxel, positioned in the centre of the proximal upper third of the semitendinosus muscle (SemiT). The SemiT muscle was selected instead of the (most frequently injured) biceps femoris long head (BF_LH_) muscle, as this resulted in better shimming results and superior field homogeneity within the voxel of interest, most likely due to the fact that the SemiT muscle consists of fewer intramuscular tendon and fascia tissues compared to the more tendinous BF_LH_, making it more suitable for spectroscopy analysis. Fibre type composition was expected to be highly similar within the same muscle group within one subject (due to the presence of an across-muscle fibre composition phenotype), which has been reported by previous research as well [10, 13].

For this scanning procedure, participants were positioned supine on the scanning table, with the feet primarily directed into the scanning entrance. The spine coil was used as the primary sender coil, while a flex coil was fixed around the back of the participant’s dominant thigh acting as a receiving coil for both T1 and H-MRS sequences. The participants were instructed to lie down as still as possible throughout the entire scanning procedure. To reduce the risk of motion artefacts, the researcher applied sand bags to fix the dominant limb in its isocentre position on the scanning table. A detailed overview of the technical particularities of the spectroscopy (PRESS) sequence can be seen in Table 2.

**TABLE 2:** MR Spectroscopy sequence (Single voxel point-resolved spectroscopy (PRESS) sequence and acquisition details.

^1^H-MRS acquisition parameters	Muscle (Semitendinosus)	Water Reference
Relaxation Time (ms)	4500	4500
Echo Time (ms)	30	30
Number of averages	64	8
Water suppression	Water Saturated	None
Spectral bandwidth (Hz)	2000	2000
Voxel size (mm^3^)	(40 × 13 × 19) = 9880	(40 × 13 × 19) = 9880

Note: ^1^H-MRS, Proton Magnetic Resonance Spectroscopy; Hz, Hertz; mm, millimeters; mm^3^, cubic millimeters; MR, Magnetic Resonance; ms, milliseconds; °, degrees

These measurements were conducted in the athlete’s dominant thigh (side preferably used to kick) only, as bilateral image acquisition was not possible due to the extent of the respective field of view (FOV) and associated issues related to coil size and scanning time. The total scan time was approximately 15 minutes, taking into account the additional time needed for optimal slice and voxel positioning and shimming (i.e. increasing the magnetic field homogeneity to increase the signal to noise ratio (SNR) and hence the resolution and quality of the spectrum).

After MR imaging, participants were submitted to a performance screening battery consisting of a repeated maximal 25 m sprint, a repeated counter movement jump (CMJ) for maximal vertical jumping height, a single leg hamstring bridge test (SLHB) for the investigation of hamstring strength endurance, as well as a maximal isokinetic strength investigation of the hamstrings using isokinetic dynamometry (IKD). Each participant was submitted to this performance screening in the same standardized sequence ((1) sprint, (2) CMJ, (3) SLHB, (4) IKD). This order was chosen to guarantee maximal explosiveness during the sprint and CMJ tests, without any influence of fatigue due to preceding strength performances. As fatigue most definitely affects maximal isokinetic muscle strength measured by means of IKD evaluation, this last performance test was proceeded by a short recovery period of 30 minutes for each participant. This was done in order to decrease and standardize the potential effect of fatigue of the IKD test results.

Prior to performance and consecutive strength analyses, participants were instructed to perform a 5 minute (min) warm-up by running at a comfortable steady pace (8–10 km/h). These performance measures were conducted indoors and participants wore indoor soccer shoes to complete these testing series.

For the 25 m sprint acceleration test, participants were instructed to cover respective distance as fast as possible, starting from a stationary position. This was repeated 6 times. Between each sprinting trial, participants were instructed to return to the starting point by jogging at a comfortable pace (6-8 km/h), after which they were told to immediately position themselves in the stationary starting position again to perform the consecutive sprint. Sprinting speed was measured using the Witty timing Gate Photocell system (Microgate, Bolzano, Italy), using 3 pairs of gates positioned at 0 m, 10 m and 25 m of the linear running trajectory. Photocells were placed at respective positions, in order to primarily track starting speed and acceleration capacity, rather than maximal sprinting speed. The protocol consisted of 6 sprints with some recovery though no actual rest in between trials, in order to match with the mechanical and metabolic demands of soccer and the associated HSI risk. Both average and minimal (i.e. best) sprint times (expressed in seconds (s)) for the 0–10 m, 10–25 m and 0–25 m distances were taken for data analysis.

Maximal vertical jumping capacity was evaluated by means of a five-fold CMJ with rapid rebound in between consecutive jumps. For this CMJ analysis, the Optogait System (Microgate, Bolzano, Italy) was used. The athletes were instructed to stand between both bars in a neutral starting position, feet placed at shoulder width and equally supporting on both legs. Afterwards, the athlete was instructed to perform 5 consecutive maximal CMJs between both bars, aiming to jump as high as possible with the legs extended in the airborne phase, landing steadily in between consecutive jumps. Both average and maximal (i.e. best) jumping heights (expressed in metres, as well as average and maximal jumping power (expressed in watts) were taken for data analysis.

Local hamstring muscle strength endurance was investigated by means of a single leg hamstring bridge test (SLHB) performed by the dominant leg, until full exhaustion was reached. Only the dominant leg was evaluated, as we only collected ^1^H-MRS data on the athlete’s dominant side as well. This test was initially described by Freckleton and colleagues and was performed using the same standardized guidelines and instructions [19]. The number of repetitions the participants were able to perform adequately (without loss of quality or control, or demonstration of compensations) until reaching full exertion was taken for data analysis.

Concentric and eccentric hamstring muscle strength features were evaluated at angular velocities of 60 and 240°/s for the concentric isokinetic sequences (5 and 10 maximal repetitions (reps) respectively), and 30 and 120°/s for the eccentric sequences (3 and 5 maximal reps respectively). Different angular velocities were used in order to investigate both features of maximal strength and explosive strength in concentric and eccentric contraction modes. All measurements were carried out on the same Biodex Isokinetic Dynamometer (Biodex 4 Pro System, Biodex Medical Systems, Inc. NY, USA). Participants were positioned on the IKD chair in a standardized sitting position with 90° of knee and hip flexion, using straps to assure a fixed testing position. The dynamometer axis was aligned with the knee axis and the lower leg was fixated accordingly. After taking care of the protocol and range of motion (ROM) settings, which allowed maximal concentric and eccentric peak torque analysis throughout a fixed knee flexion-extension testing range of 100°, lower leg weight was measured for gravity correction. Consecutively, isokinetic strength testing for the different contraction modes and angular velocities was performed, in which each participant was allowed three practice trials per strength testing condition prior to actual isokinetic strength evaluation. As this study intended to assess the association between hamstring muscle fibre type dominance and isokinetic strength profile, this isokinetic strength testing procedure was only carried out for the participant’s dominant leg. For each one of the isokinetic testing protocols mentioned above, average hamstring peak torque/bodyweight (PT/BW; expressed in Nm/kg), and average time to peak torque (TPT; expressed in ms), were taken for statistical analysis. These particular outcome measures were chosen to investigate both maximal (average peak torque) and explosive (time to peak torque) muscle strength capacity.

To quantify the SemiT muscle’s carnosine content, the integral of the C2-H peak (at ~8 ppm) was quantified relative to the water peak integral (at 4.7 ppm), multiplied by 1 000 000, and calculated as arbitrary units [14, 20]. Given the quite high variability in hamstring muscle fibre type in male athletes, and in order to allow subsequent categorization of data in order to identify the participants’ hamstrings as being predominantly slow twitch, intermediate type or fast twitch, Z-scores were calculated using mean and standard deviations of the above-mentioned carnosine concentration outcomes, based on the normal distribution. For methodological details we refer to previous work [12, 21–22].

Because of poor quality of some of the carnosine and water spectra collected by means of H-MRS, data on carnosine content of 5 of the 44 participants were not taken for statistical analysis (2 participants in control and 3 participants in the HSIH group).

For each of the continuous and dependent outcome parameters, descriptive statistics were explored and data distributions were checked for normality. The primary outcome measure was the potential difference in relative SemiT carnosine contents between groups (HSI history versus matched controls), for which Student’s independent t-testing was performed. This possible association was also investigated by means of chi-square analysis, using the carnosine concentration’s derived Z-scores, to compare to what extent the cell count representation in the slow twitch, intermediate type and fast twitch muscle type groups differed significantly between the HSIH group and the control group. Participants were estimated to have predominantly (1) slow twitch, (2) intermediate type or (3) fast twitch fibre typologies if their Z-scores were (1) < -.05, (2) > -0.05 and < 0.05, and (3) > 0.05, respectively. Effect sizes for between-group differences were quantified using post-hoc Bonferroni corrections in these chi-square procedures.

After this HSIH based carnosine content comparison, correlation and linear regression analyses were performed in order to quantify potential associations between the hamstring muscle’s relative carnosine content, functional performance and isokinetic hamstring strength features (secondary outcome measure). These associations were examined with Pearson’s correlation coefficient (r) and ANOVA hypothesis tests.

All statistical procedures were carried out within the interface of SPSS Statistics Version 25 (IBM SPSS Statistics 25). Significance levels were set at α = 0.05.

## RESULTS

Between-group comparison in terms of demographics revealed that age differed significantly between groups (means and standard deviations (SD) of 22 ± 1.57 and 24.95 ± 3.47 years in control and HSIH groups, respectively, p = 0.001), while height (means and SDs of 1.81 ± 0.06 and 1.81 ± 0.06 m in control and HSIH groups, respectively, p = 0.76) and bodyweight (means and SDs of 74.89 ± 6.89 and 78.32 ± 8.80 kg in control and HSIH groups, respectively, p = 0.17) were very similar. Age was demonstrated not to be related to carnosine content within this sample of amateur soccer payers (r = 0.13, p = 0.43).

**FIG. 1:**
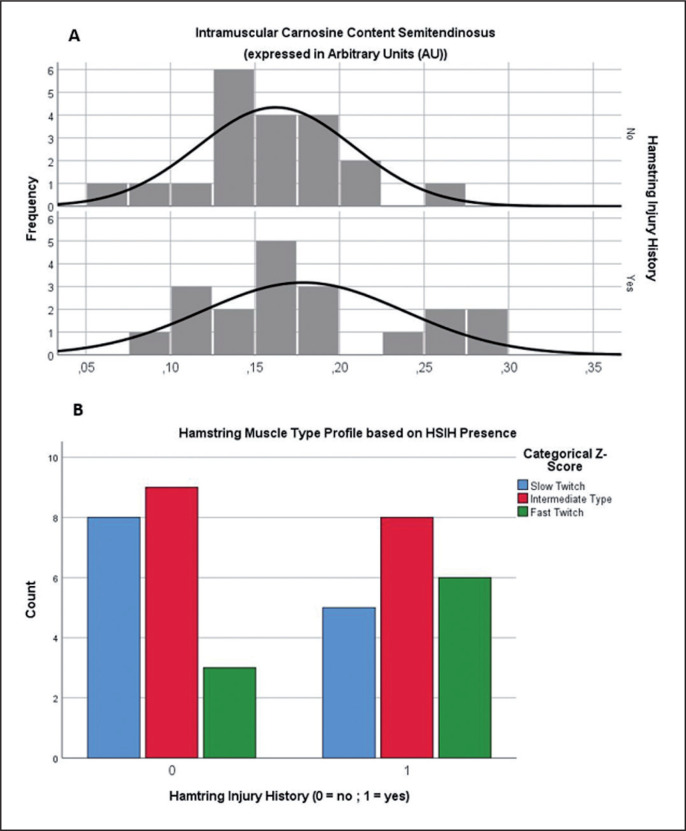
**A)** Data distribution of the relative carnosine content variable in both the control and HIH groups. Z-score transformation was used in order to categorize players in the fast twitch (Z > 0.5), intermediate type (0.5 < Z > -0.5) or slow twitch (Z < -0.5) hamstring muscle fibre type groups. **B)** Distribution of 3 major Z-score groups within both the control and HSIH groups. Chi-square testing revealed no significant differences in categorical Z-score counts based on HSIH presence. (p = 0.21). Note: Std. Dev., standard deviation; N, sample size; HIH, hamstring injury history; HSIH, hamstring strain injury history; %, percentage.

Between-group comparison revealed no differences in relative carnosine content (expressed in arbitrary units [(C2-H peak/water peak)*1000000]) or associated Z-score, either for the continuous (p = 0.34) or categorical statistical analyses (p = 0.21). The data distribution of this relative carnosine concentration parameter, used to make estimations as regards fibre type, presented a remarkably high variability in both the control and the HSIH groups (coefficient of variation (CV) = 31.2%) (Figure 1). Table 3 demonstrates the fibre type distribution within the entire sample, without making further subdivision based on HSIH.

**TABLE 3:** Fiber type distribution within entire study sample.

	Sample of Male Amateur SOCCER Players (n = 39)
Slow twitch fiber dominant (z < -0.5) (count (%))	13 (33)
Intermediate type dominant (-0.5 < z > 0.5) (count (%))	17 (44)
Fast twitch fiber dominant (z > 0.5) (count (%))	9 (23)

Note: n, study sample; %, percentage. Z < -0.5 = predominantly Slow Twitch Fibers; 0.5 < Z > -0.5 = Intermediate Type; Z > 0.5 = Predominantly Fast Twitch Fibers.

Correlation and chi-square analyses in the search of any associations between the hamstring muscle’s fibre typology and the functional performance characteristics revealed no associations between the intramuscular carnosine content and either the SLHB, maximal sprint acceleration or CMJ capacity within this sample. This was the case for correlation analysis taking into account the carnosine concentration as a continuous parameter, as well as for the ANOVA analysis assessing possible differences in performance estimates based on fibre type category. (Table 4)

**TABLE 4:** Performance outcomes in function of Fiber Type Category for SLHB, Sprint, Vertical Jump and Isokinetic Strength analyses with accompanying levels of significance (in-between-group differences using ANOVA and post hoc Bonferonni hypothesis tests).

	Slow Twitch (ST) (n = 13)	Intermediate Type (IT) (n = 17)	Fast Twitch (FT) (n = 9)	ANOVA’s p	Bonferonni’s p
Single Leg Hamstring Bridge (dominant leg) (number of reps)	36 ± 14	30 ± 6	31 ± 13	0.353	/
Average sprint time 0–10m (s)	1.86 ± 0.11	1.81 ± 0.08	1.87 ± 0.05	0.105	/
Minimal sprint time 0–10m (s)	1.77 ± 0.10	1.75 ± 0.06	1.80 ± 0.08	0.310	/
Average sprint time 10–25m (s)	2.00 ± 0.12	2.00 ± 0.09	2.05 ± 0.06	0.414	/
Minimal sprint time 10–25m (s)	1.91 ± 0.10	1.93 ± 0.07	1.97 ± 0.07	0.196	/
Average sprint time 0–25m (s)	3.86 ± 0.22	3.81 ± 0.15	3.92 ± 0.09	0.257	/
Minimal sprint time 0–25m (s)	3.69 ± 0.19	3.70 ± 0.11	3.79 ± 0.12	0.230	/
Average vertical jumping height (m)	40.12 ± 5.72	37.61 ± 5.42	37.50 ± 3.70	0.384	/
Maximal vertical jumping height (m)	40.82 ± 8.69	40.56 ± 6.03	39.79 ± 4.69	0.940	/
Average vertical jumping power (W)	22.98 ± 5.05	19.94 ± 3.25	21.21 ± 3.06	0.139	/
Maximal vertical jumping power (W)	26.11 ± 8.30	20.90 ± 3.50	22.64 ± 3.69	0.061	0.058 (ST vs IT)
Average PT/BW Hamstrings Con @60°/s (Nm/kg)	1.35 ± 0.26	1.38 ± 0.30	1.39 ± 0.28	0.956	/
TPT Hamstrings Con @60°/s (ms)	547 ± 124	557 ± 164	619 ± 235	0.602	/
APT Hamstrings Con @60°/s (°)	53 ± 10	57 ± 13	56 ± 16	0.656	/
Average Power Hamstrings Con @60°/s (W)	80.3 ± 12.8	80.5 ± 21.2	76.6 ± 21.9	0.873	/
TW/BW Hamstrings Con @60°/s (J/kg)	7.99 ± 1.79	8.39 ± 2.51	7.52 ± 1.68	0.610	/
Average PT/BW Hamstrings Con @240°/s (Nm/kg)	0.84 ± 0.19	0.85 ± 0.19	1.01 ± 0.22	0.119	/
TPT Hamstrings Con @240°/s (ms)	244 ± 38	199 ± 57	188 ± 50	0.025	0.044 (ST vs FT)
APT Hamstrings Con @240°/s (°)	64 ± 7	58 ± 11	52 ± 16	0.081	0.084 (ST vs FT)
Average Power Hamstrings Con @240°/s (W)	111.7 ± 21.0	121.5 ± 31.0	132.9 ± 36.3	0.283	/
TW/BW Hamstrings Con @240°/s (J/kg)	8.19 ± 2.04	8.59 ± 2.40	9.38 ± 2.35	0.496	/
Average PT/BW Hamstrings Ecc @30°/s (Nm/kg)	2.15 ± 0.41	2.21 ± 0.35	2.20 ± 0.57	0.937	/
TPT Hamstrings Ecc @30°/s (ms)	2737 ± 319	2741 ± 318	2735 ± 0.57	0.993	/
APT Hamstrings Ecc @30°/s (°)	21 ± 9	23 ± 10	20 ± 6	0.668	/
Average Power Hamstrings Ecc @30°/s (W)	60.0 ± 8.1	65.2 ± 12.3	60.0 ± 13.6	0.397	/
TW/BW hamstrings Ecc @30°/s (J/kg)	520.1 ± 79.6	567.5 ± 137.6	495.1 ± 128.4	0.478	/
Average PT/BW Hamstrings Ecc @120°/s (Nm/kg)	1.80 ± 0.32	1.81 ± 0.46	1.94 ± 0.58	0.750	/
TPT Hamstrings Ecc @120°/s (ms)	673 ± 108	649 ± 97	646 ± 114	0.794	/
APT Hamstrings Ecc @120°/s (°)	29 ± 11	32 ± 12	32 ± 15	0.703	/
Average Power Hamstrings Ecc @120°/s (W)	151.2 ± 35.6	177.9 ± 37.6	163.8 ± 45.6	0.212	/
TW/BW Hamstrings Ecc @120°/s (J/kg)	8.33 ± 2.00	9.23 ± 2.08	9.33 ± 2.90	0.508	/

Note: APT, Angle of Peak Torque; BW, Body Weight; Con, Concentric; Ecc, Eccentric; J, Joule; kg, kilogram; m, meters; m, meters; ms, milliseconds; n, sample size; Nm, Newton meter; p, probability; PT, Peak Torque; reps, repetitions; s, seconds; TPT, Time to Peak Torque; TW, Total Work; vs, versus; W, Watts; °, degrees.

Correlation analysis did not reveal any associations between relative carnosine content and isokinetic thigh strength, whereas the ANOVA analyses (hamstring strength as a function of categorized Z-cores) did reveal a significant between-group difference for the hamstring’s time to peak torque measured concentrically at a 240°/s angular velocity (p = 0.025). The FT dominant participants demonstrated the shortest TPT and the ST dominant participants presenting the longest TPT, with the intermediate type participants scoring somewhere in between. Post hoc pairwise comparisons with Bonferroni correction revealed that this difference was located between the ST and FT fibre category groups, presenting times to peak torque of 244 ± 38 ms and 188 ± 50 ms, respectively (p = 0.044) (Table 4).

## DISCUSSION

This is the first study investigating hamstring muscle fibre typology non-invasively by means of ^1^H-MRS, in function of both HSIH and performance in a sample of male soccer players. The results revealed that the hamstring’s carnosine content, investigated in the proximal part of the SemiT, was highly variable in male amateur soccer players and did not present a particularly uniform carnosine concentration but a rather large inter-individual variability, indicative for the absence of distinctly predominant fibre typology. Interestingly, this carnosine concentration did not present any association with HSIH, nor with any of the functional performance-related outcomes. Only the TPT of the hamstring muscles measured concentrically at 240°/s presented a significant association with muscle fibre typology, as it was found to be significantly shorter in players with a fast twitch dominant fibre type, compared to players with a slow twitch fibre dominance.

The first important finding of this study is that the hamstring muscle group of this study sample of amateur soccer players presented high inter-individual variability in intramuscular carnosine content. This carnosine content distribution indicates that the hamstring muscles within this study sample present a rather intermediate fibre typology profile and can probably not be considered a fast twitch fibre dominant muscle group. These findings are in contrast with what has been stated in previous literature, i.e. that the hamstrings mainly consist of fast twitch fibres (ca. 55%), making them particularly vulnerable to eccentric induced muscle damage (EIMD) and fatigue, and therefore highly susceptible to strain injury in explosive field sports exposing them to repeated bouts of high speed running [5, 7, 10, 23]. Albeit valuable to provide some kind of a normative database, this theoretical framework has very little to do with the actual HSI risk profile in soccer, as it was composed based on in vitro research on necrotic hamstring muscle tissue in cadavers [10]. In their study, Garret and colleagues explored hamstring muscle fibre composition in 7 different sites across the hamstring muscle unit, which revealed a type II (FT) muscle fibre representation of 55% and 54% in the proximal and distal regions of the BFLH, 55% and 60% in the proximal and distal regions of the SemiT, and 51% and 51% in the proximal and distal regions of the semimembranosus, respectively. Based on these findings, together with a relevant preliminary cadaver study and referred work [5, 7], we are not able to make estimations on the role of fibre type composition in the hamstring’s strain injury risk as the research investigated metabolically inactive muscle tissue in the elderly, without any association with the soccer related loading mechanism or hamstring injury occurrence. Nonetheless, it provided us with insights as regards the (varying) profile of the distribution with the bi-articular hamstring muscle unit, and provided scientific evidence for our methodological approach selecting a voxel in the proximal SemiT for ^1^H-MRS measurements, as the fibre distribution at this particular location was established to be highly similar to the one in the (proximal) BFLH region. The present results should be interpreted carefully and do not allow us to use proximal SemiT carnosine content identification for general hamstring muscle group fibre type determination. Future studies should include voxel selection in proximal, mid-section and distal hamstring muscle regions as well. Interestingly, however, the results of the present study are in line with the findings of recent in vivo work of Evangelidis and colleagues [24], who evaluated BF_LH_ fibre distribution (by means of sodium dodecyl sulfate-polyacrylamide gel electrophoresis (SDS-PAGE)), muscle volume and maximal and explosive knee flexor strength in a sample of 31 healthy male volunteers (non-athletic). They found a balanced muscle fibre composition in the BF_LH_, consisting of 47% type I (slow twitch) muscle fibres, 36% type IIa (intermediate twitch) muscle fibres and 17% type IIx muscle fibres (fast twitch). Beside this renewing insight, they also found no association between muscle fibre type and maximal or explosive knee flexor strength. In their study, the large inter-individual variation in fibre type most possibly has an important share in these findings, which is most likely to be the case for the present study findings as well. Indeed, Our study results indicated that the hamstring muscle’s relative carnosine content demonstrated a large CV (31.2%) as well, implying a large spread and heterogeneity in muscle fibre type composition within this study’s sample of amateur soccer players. This large CV probably co-explains the absence of associations with HSIH and performance (as is the case for the above-mentioned study as well) [24], particularly given the fact that the selected study sample was rather limited in size and consisted of amateur athletes rather than professionals. The sample of Evangelidis consisted of recreationally active men, while ours consisted of amateur male soccer players. Evangelidis et al. concluded that the BF_LH_ should not be considered a fast twitch muscle fibre dominant muscle entity, and that its fibre composition is probably not part of its particular vulnerability to strain injury in their particular study sample.

The second and most important finding of this study is that estimated hamstring muscle fibre typology in amateur male soccer players did not present any association with HSIH. This is the opposite of what was expected based on existing theoretical frameworks [7, 10], and recently published work on the association between calf muscle carnosine content and hamstring strain injury risk [12]. These authors used the same ^1^H-MRS procedure to determine the intramuscular carnosine content using the uni-articular soleus muscle instead of the hamstrings in a cohort of professional male soccer players who were prospectively monitored for hamstring injury occurrence over a period of 3 consecutive competition seasons. Similarly to our study, they established that male professional soccer players exhibit a widely varying muscle fibre composition (44% slow twitch, 40% intermediate type and 15% fast twitch). Contrary to our findings, however, the results of their prospective analysis did indicate that athletes with a fast typology had a 5-fold higher risk of sustaining an index HSI compared to athletes with a predominantly slow twitch fibre profile. Although these findings seem conflicting, they are most likely to be explained by the essential difference in study samples. Lievens et al. assessed a cohort of professional male soccer players, who are equally exposed to highly strict and standardized training and competition sessions throughout a professionally composed competition season. Our sample consisted of amateur male soccer players, for whom soccer exposure by means of training and match participation is known to differ essentially between clubs and individual players. Moreover, other features such as weekly diet, weekly activity levels and participation in other recreational sports will influence the amateur athlete’s injury risk and performance profile probably to a higher extent than the player’s muscle fibre content, which might be slightly dependent on these differential soccer exposure and general behavioural features as well. We suggest that in amateur sports, too many other factors influence the player’s injury risk and performance capacity due to the relatively low exposure rates throughout a competition season and large variety in behaviour outside of soccer specific training and competition times. Another, probably less pivotal, reason for the different findings in the study of Lievens et al. is that these authors particularly wanted to identify whether the overall (muscle group-independent) skeletal muscle fibre typology (in professional male soccer players) assessed in the soleus muscle was related to hamstring strain injury susceptibility. Our study specifically wanted to verify to what extent the hamstring’s proper muscle fibre composition should be held accountable for the unremittingly high hamstring strain injury incidence (in amateur soccer players). The authors of the present study suggest that the fact that Lievens et al. found FT dominant players to be more susceptible to HSI might presumably partly be explained by the fact that these players possess more explosiveness, agility and speed, increasing the amount of mechanical strain imposed on the hamstrings during sport-specific manoeuvres. In accordance with this line of reasoning, the authors also suggested that players with a fast twitch dominant calf-muscle profile might present a higher HSI injury risk due to lower fatigue tolerance and the detrimental influence of fatigue on neuromuscular coordination capacity, potentially putting the hamstrings at increased risk for strain injury during high load lengthening contractions as is the case in high speed running. The present study did not find any associations between ^1^H-MRS-estimated hamstring muscle fibre typology and HSIH. Nevertheless, a higher percentage of fast twitch fibres in the hamstring muscle of the individual amateur soccer player might predispose his hamstrings relatively more to HSI occurrence, but there is currently no evidence to state that this trend applies to the hamstring muscle unit as such. It is also important to note that within the population of recreational athletes, significant associations between maximal and explosive knee flexor strength and muscle thickness (objectified by means of T1 magnetic resonance imaging) were identified [26, 30]). Other features of muscle architecture (e.g. muscle thickness and fascicle length) are therefore more likely to have an influence on the HSI risk in the recreationally active population, as these features significantly related to strength performance capacity as well [24, 26].

As regards our third and final hypothesis, no clear associations could be established between hamstring muscle fibre type and functional performance or isokinetic strength profile. Data analysis only established a significant association between the SemiT’s carnosine content and the hamstring muscle’s time to peak torque variable measured concentrically at 240°/s, which was established to be significantly shorter in participants presenting higher SemiT carnosine contents. Given this was the first study assessing the association between hamstring muscle fibre type and performance in a population of amateur soccer players, comparison with existing research is impossible. Albeit not in a sample of football players, the results of Evangelidis and colleagues are in line with our findings, demonstrating no association between maximal or explosive hamstring muscle strength and fibre typology [24]. Other fairly recent ^1^H-MRS based fibre type determination studies assessing the relationship between intramuscular calf muscle carnosine content and athletic performance did find some significant associations between some particular speed-related performance characteristics and higher Z-scores/carnosine contents, albeit to a limited extent [17–18, 25]. The fact that we did not find any association between muscle fibre typology and performance, whereas previous work using almost the same methodological approach did, might again be the consequence of the differences between amateur and professional athletes. Athletic performance, in particular explosiveness, is dependent on many more factors (e.g. neuromuscular control, muscle strength, plyometric capacity, reaction speed) than muscle fibre typology alone. As these others features are adequately and highly similarly trained in professional athletes, the effect of muscle fibre type on athletic performance is considered to be much more important in professional compared to amateur athletes. Professional athletes are exposed to the same athletic exposure and possess similar motor skills, trained at an almost identical level (within the same sports domain). This is not the case for amateur athletes, where performance is expected to be primarily dependent on (1) physical fitness, (2) sports exposure, diet and behaviour, and (3) medical history and sports experience, with the underlying muscle fibre type only being a small part of his performance capacity profile. Beside differing athletic profiles between amateur and elite players, differences between carnosine content in SemiT and soleus muscle may play a role in finding different results here as well. Therefore, future work should ideally look into associations between fibre typology, injury susceptibility and performance, assessing different muscle groups and several intramuscular locations within those muscles.

As far as the role of hamstring muscle fibre type is concerned, the authors would not recommend further studies on its value in sport-specific injury risk in amateur athletes. The present results suggest that in amateur soccer players, hamstring muscle fibre type demonstrates very high inter-individual variability and is not related to performance, and can therefore probably not be considered a strong risk factor for running-related hamstring strain injuries in the recreationally active population.

### Strengths and Limitations

Despite being the very first study assessing the role of hamstring muscle fibre type composition in function of HSIH and performance in male soccer players, it is not without limitations. First, this was a retrospective study and therefore the present results do not allow the identification of any cause-effect relationships as regards the role of muscle fibre type in strain injury vulnerability. Second, our sample size was fairly limited. Given the high variability in carnosine content established within our selection of male soccer players, a bigger sample might have augmented the sensitivity and specificity of our findings (certainly as we had to exclude carnosine-content related data of 5 of the 44 participants – 3 in the HSIH group and 2 in the control group). We based our sample size on the ones documented in previous work using ^1^H-MRS for the quantification of intramuscular carnosine contents in athletes for fibre type determination [14]. Using the effect sizes documented in the respective study, power and associated sample size calculations demonstrated a power-associated β-value of at least 0.80, using an average participant count of n = 17 in each of the included groups (total sample count of n = 34). Given the fact that these authors worked with more distinct metabolic athletic profiles (sprinters, marathon runners and athletes with a mixed explosive/endurance loading profile) [14], CV of the carnosine contents are assumed to be lower within athletic groups, allowing lower effect sizes to make valid conclusions. Given the fact that soccer players are repeatedly exposed to a mix of explosive and endurance related loading efforts, they are expected to present less distinct metabolic muscle fibre type features and a CV as regards their carnosine concentrations was to be expected. Future research investigating muscle fibre composition using ^1^H-MRS in athletic populations with mixed metabolic loading profiles, might therefore benefit from larger sample sizes. Third, H-MRS analysis was performed selecting a standardized muscle voxel positioned centrally (transverse plane) in the proximal third (coronal and sagittal planes) of the SemiT in the dominant (i.e. preferred kicking) leg of the participants. We chose to do so because we wanted to (1) avoid biased results based on the factor dominance and (2) maximize the ^1^H-MRS spectra’s quality and improve the results of the shimming procedure to allow maximal magnetic field homogeneity within the voxel of interest (pilot investigations on the most frequently injured BF_LH_ revealed issues in the shimming procedure, making correct spectroscopy measurements and interpretation of these results hardly feasible). Taking into account the side and muscle location subject to the last HSI in the HSIH group within the voxel selection for consecutive spectroscopy analysis (and choosing similar muscle locations in the control group) might have resulted in different findings and insights (although previous work demonstrated muscle fibre composition to be identical when comparing both limbs) [27]. The authors are however fairly certain to have opted for the best approach in terms of research quality and associated validity and reliability, as opting to include differential muscle locations in both the dominant and non-dominant legs within an athletic sample already consisting of a substantial amount of inter-individual variability as far as muscle fibre architecture is concerned would make interpretation of the results very complex or even impossible. Because we also wanted to investigate the associations between hamstring fibre composition and athletic performance/strength, we are certain we made the correct choice standardizing in terms of body side and muscle location for the ^1^H-MRS procedure. Lastly, it must be acknowledged that the TPT measure is less reliable at high measuring speeds [28]. This is also why this finding was considered negligible and the authors concluded that performance presented no association with SemiT’s carnosine content.

## CONCLUSIONS

Hamstring muscle fibre composition was established to be highly variable in male amateur soccer players and presented no association with HSIH or with athletic performance evaluated by means of sprints, vertical jump or hamstring strength endurance capacity or any isokinetic hamstring strength parameters. These study findings indicate that (in male amateur soccer) (1) the hamstring muscles do not seem to have a particular fibre type dominance and present substantial inter-individual variability (2) fibre composition does not relate to hamstring injury occurrence or athletic performance or hamstring strength in amateur male soccer players. Based on the results, the authors suggest that hamstring muscle fibre type should probably not be considered a substantial risk factor contributing to the hamstrings’ susceptibility to strain injury in amateur male soccer players.

### Practical implications

– Hamstring muscle fibre type, evaluated in the proximal part of the semitendinosus muscle, is not associated with their strain injury risk or athletic performance in amateur male soccer players.
– In amateur sports, many other confounding factors are believed to influence the player’s performance capacity and injury risk profile. These factors are mostly related to large variation in athletic behaviour and background (both medical and sports related) at the recreational level of soccer competition.
– There is no need to focus on potential differences in muscle fibre type when wanting to organize muscle injury prevention at the amateur level.
– The large variability in fibre typology in between amateur soccer players indicates that recovery sessions should ideally be organized at the level of the individual athlete (and his typology), as generic recovery content can questionably sufficiently address both the fast and slow twitch dominant players.
